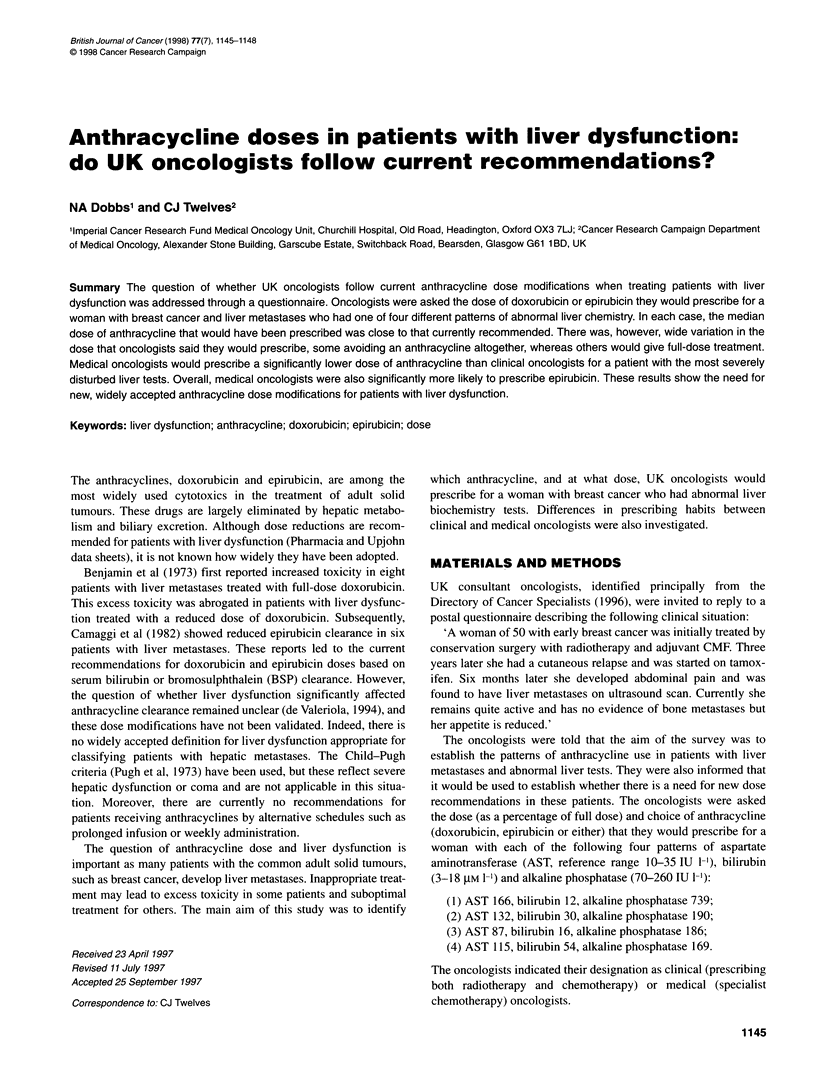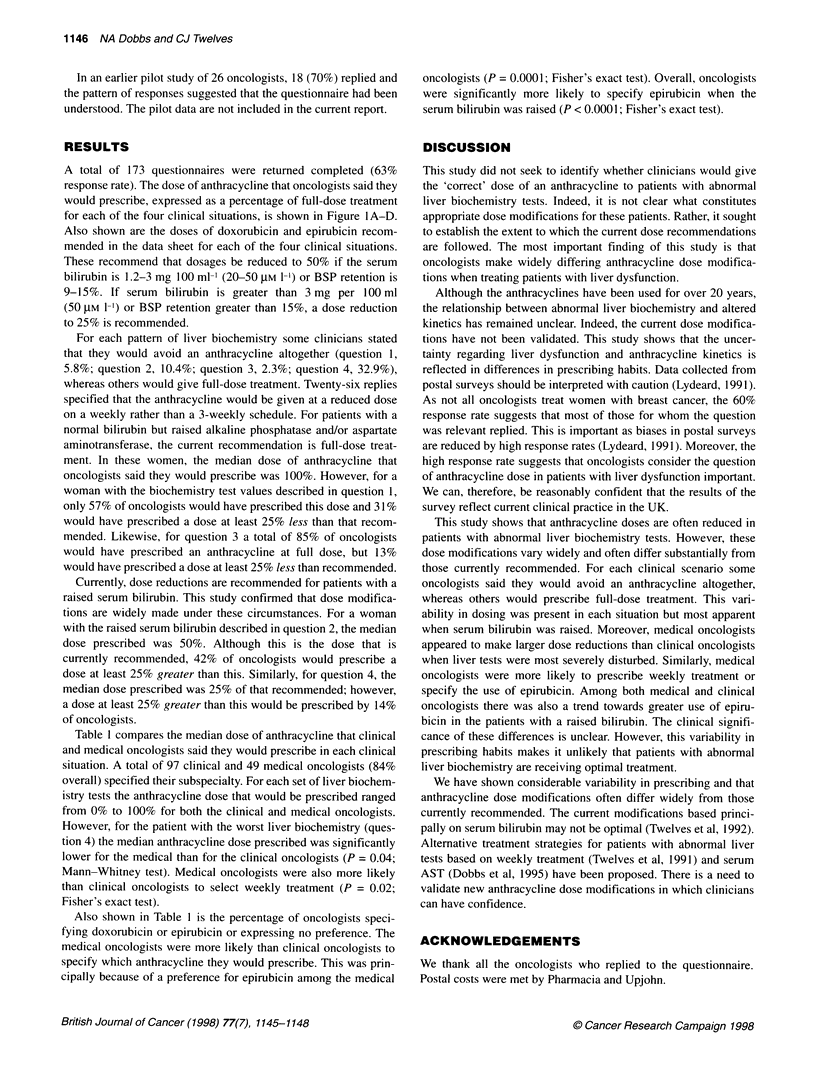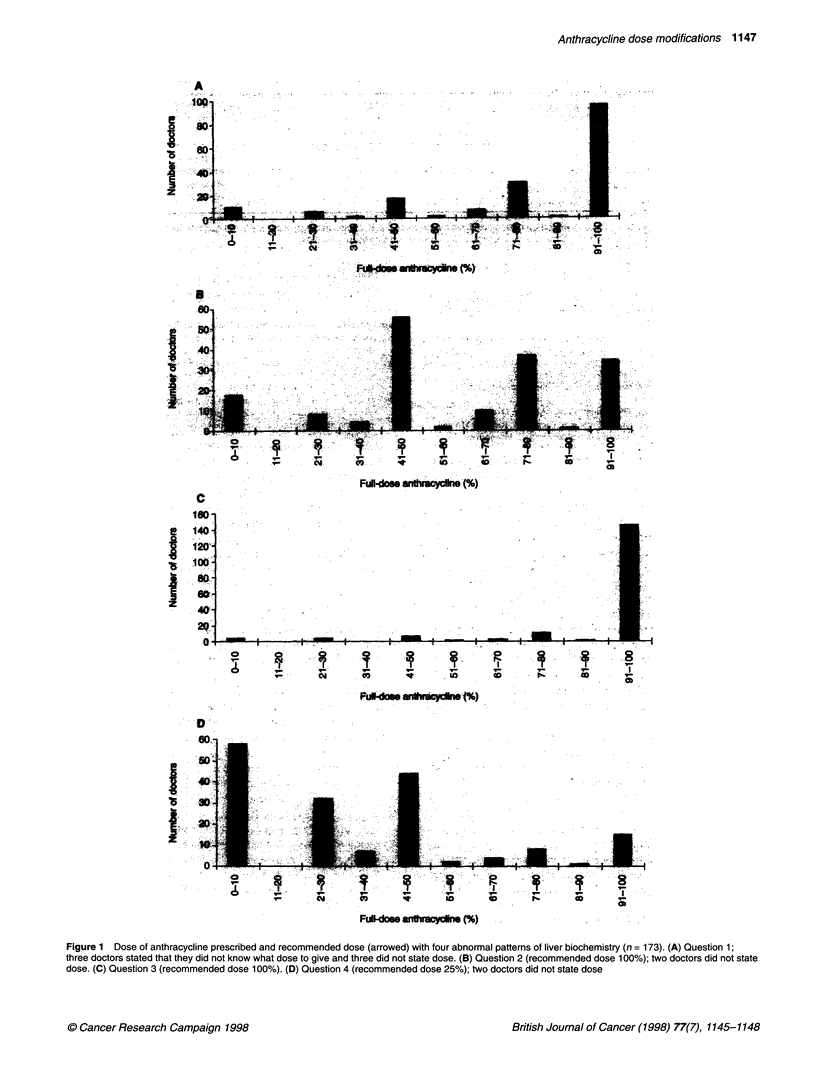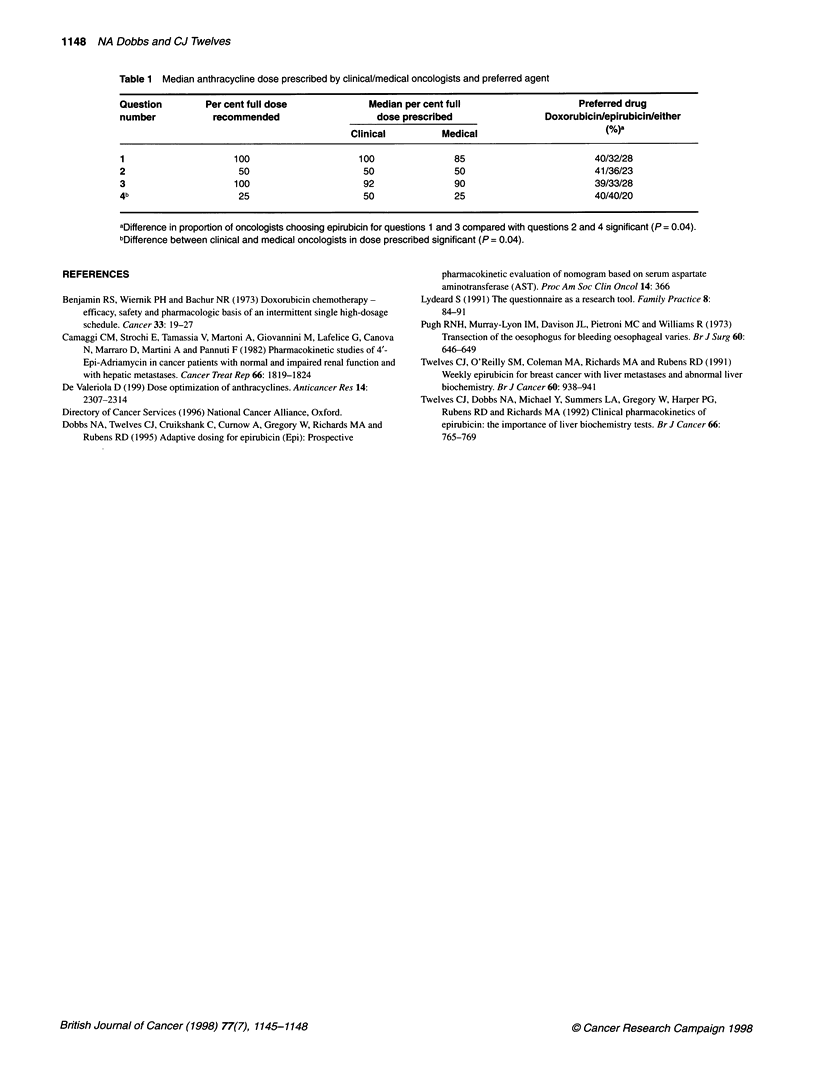# Anthracycline doses in patients with liver dysfunction: do UK oncologists follow current recommendations?

**DOI:** 10.1038/bjc.1998.190

**Published:** 1998-04

**Authors:** N. A. Dobbs, C. J. Twelves

**Affiliations:** Imperial Cancer Research Fund Medical Oncology Unit, Churchill Hospital, Headington, Oxford, UK.

## Abstract

The question of whether UK oncologists follow current anthracycline dose modifications when treating patients with liver dysfunction was addressed through a questionnaire. Oncologists were asked the dose of doxorubicin or epirubicin they would prescribe for a woman with breast cancer and liver metastases who had one of four different patterns of abnormal liver chemistry. In each case, the median dose of anthracycline that would have been prescribed was close to that currently recommended. There was, however, wide variation in the dose that oncologists said they would prescribe, some avoiding an anthracycline altogether, whereas others would give full-dose treatment. Medical oncologists would prescribe a significantly lower dose of anthracycline than clinical oncologists for a patient with the most severely disturbed liver tests. Overall, medical oncologists were also significantly more likely to prescribe epirubicin. These results show the need for new, widely accepted anthracycline dose modifications for patients with liver dysfunction.


British Journal of Cancer (1998) 77(7), 1145–1148
© 1998 Cancer Research Campaign

### Anthracycline doses in patients with liver dysfunction: do UK oncologists follow current recommendations?

**NA Dobbs[1] and CJ Twelves[2]**

[1]Imperial Cancer Research Fund Medical Oncology Unit, Churchill Hospital, Old Road, Headington, Oxford OX3 7LJ; [2]Cancer Research Campaign Department of Medical Oncology, Alexander Stone Building, Garscube Estate, Switchback Road, Bearsden, Glasgow G61 1BD, UK

**Summary** The question of whether UK oncologists follow current anthracycline dose modifications when treating patients with liver dysfunction was addressed through a questionnaire. Oncologists were asked the dose of doxorubicin or epirubicin they would prescribe for a woman with breast cancer and liver metastases who had one of four different patterns of abnormal liver chemistry. In each case, the median dose of anthracycline that would have been prescribed was close to that currently recommended. There was, however, wide variation in the dose that oncologists said they would prescribe, some avoiding an anthracycline altogether, whereas others would give full-dose treatment. Medical oncologists would prescribe a significantly lower dose of anthracycline than clinical oncologists for a patient with the most severely disturbed liver tests. Overall, medical oncologists were also significantly more likely to prescribe epirubicin. These results show the need for new, widely accepted anthracycline dose modifications for patients with liver dysfunction.

**Keywords:** liver dysfunction; anthracycline; doxorubicin; epirubicin; dose

The anthracyclines, doxorubicin and epirubicin, are among the most widely used cytotoxics in the treatment of adult solid tumours. These drugs are largely eliminated by hepatic metabolism and biliary excretion. Although dose reductions are recommended for patients with liver dysfunction (Pharmacia and Upjohn data sheets), it is not known how widely they have been adopted.

Benjamin et al (1973) first reported increased toxicity in eight patients with liver metastases treated with full-dose doxorubicin. This excess toxicity was abrogated in patients with liver dysfunction treated with a reduced dose of doxorubicin. Subsequently, Camaggi et al (1982) showed reduced epirubicin clearance in six patients with liver metastases. These reports led to the current recommendations for doxorubicin and epirubicin doses based on serum bilirubin or bromosulphthalein (BSP) clearance. However, the question of whether liver dysfunction significantly affected anthracycline clearance remained unclear (de Valeriola, 1994), and these dose modifications have not been validated. Indeed, there is no widely accepted definition for liver dysfunction appropriate for classifying patients with hepatic metastases. The Child–Pugh criteria (Pugh et al, 1973) have been used, but these reflect severe hepatic dysfunction or coma and are not applicable in this situation. Moreover, there are currently no recommendations for patients receiving anthracyclines by alternative schedules such as prolonged infusion or weekly administration.

The question of anthracycline dose and liver dysfunction is important as many patients with the common adult solid tumours, such as breast cancer, develop liver metastases. Inappropriate treatment may lead to excess toxicity in some patients and suboptimal treatment for others. The main aim of this study was to identify which anthracycline, and at what dose, UK oncologists would prescribe for a woman with breast cancer who had abnormal liver biochemistry tests. Differences in prescribing habits between clinical and medical oncologists were also investigated.

*Received 23 April 1997*
*Revised 11 July 1997*
*Accepted 25 September 1997*

*Correspondence to:* CJ Twelves

### MATERIALS AND METHODS

UK consultant oncologists, identified principally from the Directory of Cancer Specialists (1996), were invited to reply to a postal questionnaire describing the following clinical situation:

'A woman of 50 with early breast cancer was initially treated by conservation surgery with radiotherapy and adjuvant CMF. Three years later she had a cutaneous relapse and was started on tamoxifen. Six months later she developed abdominal pain and was found to have liver metastases on ultrasound scan. Currently she remains quite active and has no evidence of bone metastases but her appetite is reduced.'

The oncologists were told that the aim of the survey was to establish the patterns of anthracycline use in patients with liver metastases and abnormal liver tests. They were also informed that it would be used to establish whether there is a need for new dose recommendations in these patients. The oncologists were asked the dose (as a percentage of full dose) and choice of anthracycline (doxorubicin, epirubicin or either) that they would prescribe for a woman with each of the following four patterns of aspartate aminotransferase (AST, reference range 10–35 IU $l^{-1}$), bilirubin (3–18 $\mu M$ $l^{-1}$) and alkaline phosphatase (70–260 IU $l^{-1}$):

(1) AST 166, bilirubin 12, alkaline phosphatase 739;
(2) AST 132, bilirubin 30, alkaline phosphatase 190;
(3) AST 87, bilirubin 16, alkaline phosphatase 186;
(4) AST 115, bilirubin 54, alkaline phosphatase 169.

The oncologists indicated their designation as clinical (prescribing both radiotherapy and chemotherapy) or medical (specialist chemotherapy) oncologists.

In an earlier pilot study of 26 oncologists, 18 (70%) replied and the pattern of responses suggested that the questionnaire had been understood. The pilot data are not included in the current report.

### RESULTS

A total of 173 questionnaires were returned completed (63% response rate). The dose of anthracycline that oncologists said they would prescribe, expressed as a percentage of full-dose treatment for each of the four clinical situations, is shown in Figure 1A–D. Also shown are the doses of doxorubicin and epirubicin recommended in the data sheet for each of the four clinical situations. These recommend that dosages be reduced to 50% if the serum bilirubin is 1.2–3 mg 100 $ml^{-1}$ (20–50 μM $l^{-1}$) or BSP retention is 9–15%. If serum bilirubin is greater than 3 mg per 100 ml (50 μM $l^{-1}$) or BSP retention greater than 15%, a dose reduction to 25% is recommended.

For each pattern of liver biochemistry some clinicians stated that they would avoid an anthracycline altogether (question 1, 5.8%; question 2, 10.4%; question 3, 2.3%; question 4, 32.9%), whereas others would give full-dose treatment. Twenty-six replies specified that the anthracycline would be given at a reduced dose on a weekly rather than a 3-weekly schedule. For patients with a normal bilirubin but raised alkaline phosphatase and/or aspartate aminotransferase, the current recommendation is full-dose treatment. In these women, the median dose of anthracycline that oncologists said they would prescribe was 100%. However, for a woman with the biochemistry test values described in question 1, only 57% of oncologists would have prescribed this dose and 31% would have prescribed a dose at least 25% *less* than that recommended. Likewise, for question 3 a total of 85% of oncologists would have prescribed an anthracycline at full dose, but 13% would have prescribed a dose at least 25% *less* than recommended.

Currently, dose reductions are recommended for patients with a raised serum bilirubin. This study confirmed that dose modifications are widely made under these circumstances. For a woman with the raised serum bilirubin described in question 2, the median dose prescribed was 50%. Although this is the dose that is currently recommended, 42% of oncologists would prescribe a dose at least 25% *greater* than this. Similarly, for question 4, the median dose prescribed was 25% of that recommended; however, a dose at least 25% *greater* than this would be prescribed by 14% of oncologists.

Table 1 compares the median dose of anthracycline that clinical and medical oncologists said they would prescribe in each clinical situation. A total of 97 clinical and 49 medical oncologists (84% overall) specified their subspecialty. For each set of liver biochemistry tests the anthracycline dose that would be prescribed ranged from 0% to 100% for both the clinical and medical oncologists. However, for the patient with the worst liver biochemistry (question 4) the median anthracycline dose prescribed was significantly lower for the medical than for the clinical oncologists ($P = 0.04$; Mann–Whitney test). Medical oncologists were also more likely than clinical oncologists to select weekly treatment ($P = 0.02$; Fisher's exact test).

Also shown in Table 1 is the percentage of oncologists specifying doxorubicin or epirubicin or expressing no preference. The medical oncologists were more likely than clinical oncologists to specify which anthracycline they would prescribe. This was principally because of a preference for epirubicin among the medical oncologists ($P = 0.0001$; Fisher's exact test). Overall, oncologists were significantly more likely to specify epirubicin when the serum bilirubin was raised ($P < 0.0001$; Fisher's exact test).

### DISCUSSION

This study did not seek to identify whether clinicians would give the 'correct' dose of an anthracycline to patients with abnormal liver biochemistry tests. Indeed, it is not clear what constitutes appropriate dose modifications for these patients. Rather, it sought to establish the extent to which the current dose recommendations are followed. The most important finding of this study is that oncologists make widely differing anthracycline dose modifications when treating patients with liver dysfunction.

Although the anthracyclines have been used for over 20 years, the relationship between abnormal liver biochemistry and altered kinetics has remained unclear. Indeed, the current dose modifications have not been validated. This study shows that the uncertainty regarding liver dysfunction and anthracycline kinetics is reflected in differences in prescribing habits. Data collected from postal surveys should be interpreted with caution (Lydeard, 1991). As not all oncologists treat women with breast cancer, the 60% response rate suggests that most of those for whom the question was relevant replied. This is important as biases in postal surveys are reduced by high response rates (Lydeard, 1991). Moreover, the high response rate suggests that oncologists consider the question of anthracycline dose in patients with liver dysfunction important. We can, therefore, be reasonably confident that the results of the survey reflect current clinical practice in the UK.

This study shows that anthracycline doses are often reduced in patients with abnormal liver biochemistry tests. However, these dose modifications vary widely and often differ substantially from those currently recommended. For each clinical scenario some oncologists said they would avoid an anthracycline altogether, whereas others would prescribe full-dose treatment. This variability in dosing was present in each situation but most apparent when serum bilirubin was raised. Moreover, medical oncologists appeared to make larger dose reductions than clinical oncologists when liver tests were most severely disturbed. Similarly, medical oncologists were more likely to prescribe weekly treatment or specify the use of epirubicin. Among both medical and clinical oncologists there was also a trend towards greater use of epirubicin in the patients with a raised bilirubin. The clinical significance of these differences is unclear. However, this variability in prescribing habits makes it unlikely that patients with abnormal liver biochemistry are receiving optimal treatment.

We have shown considerable variability in prescribing and that anthracycline dose modifications often differ widely from those currently recommended. The current modifications based principally on serum bilirubin may not be optimal (Twelves et al, 1992). Alternative treatment strategies for patients with abnormal liver tests based on weekly treatment (Twelves et al, 1991) and serum AST (Dobbs et al, 1995) have been proposed. There is a need to validate new anthracycline dose modifications in which clinicians can have confidence.

### ACKNOWLEDGEMENTS

We thank all the oncologists who replied to the questionnaire. Postal costs were met by Pharmacia and Upjohn.

© Cancer Research Campaign 1998

**Figure 1** Dose of anthracycline prescribed and recommended dose (arrowed) with four abnormal patterns of liver biochemistry ($n$ = 173). (**A**) Question 1; three doctors stated that they did not know what dose to give and three did not state dose. (**B**) Question 2 (recommended dose 100%); two doctors did not state dose. (**C**) Question 3 (recommended dose 100%). (**D**) Question 4 (recommended dose 25%); two doctors did not state dose

**Table 1** Median anthracycline dose prescribed by clinical/medical oncologists and preferred agent

| Question number | Per cent full dose recommended | Median per cent full dose prescribed | | Preferred drug Doxorubicin/epirubicin/either (%)[a] |
|---|---|---|---|---|
| | | Clinical | Medical | |
| 1 | 100 | 100 | 85 | 40/32/28 |
| 2 | 50 | 50 | 50 | 41/36/23 |
| 3 | 100 | 92 | 90 | 39/33/28 |
| 4[b] | 25 | 50 | 25 | 40/40/20 |

[a]Difference in proportion of oncologists choosing epirubicin for questions 1 and 3 compared with questions 2 and 4 significant ($P = 0.04$).
[b]Difference between clinical and medical oncologists in dose prescribed significant ($P = 0.04$).

### REFERENCES

Benjamin RS, Wiernik PH and Bachur NR (1973) Doxorubicin chemotherapy – efficacy, safety and pharmacologic basis of an intermittent single high-dosage schedule. *Cancer* **33**: 19–27

Camaggi CM, Strochi E, Tamassia V, Martoni A, Giovannini M, Lafelice G, Canova N, Marraro D, Martini A and Pannuti F (1982) Pharmacokinetic studies of 4′-Epi-Adriamycin in cancer patients with normal and impaired renal function and with hepatic metastases. *Cancer Treat Rep* **66**: 1819–1824

De Valeriola D (199) Dose optimization of anthracyclines. *Anticancer Res* **14**: 2307–2314

Directory of Cancer Services (1996) National Cancer Alliance, Oxford.

Dobbs NA, Twelves CJ, Cruikshank C, Curnow A, Gregory W, Richards MA and Rubens RD (1995) Adaptive dosing for epirubicin (Epi): Prospective pharmacokinetic evaluation of nomogram based on serum aspartate aminotransferase (AST). *Proc Am Soc Clin Oncol* **14**: 366

Lydeard S (1991) The questionnaire as a research tool. *Family Practice* **8**: 84–91

Pugh RNH, Murray-Lyon IM, Davison JL, Pietroni MC and Williams R (1973) Transection of the oesophogus for bleeding oesophageal varies. *Br J Surg* **60**: 646–649

Twelves CJ, O'Reilly SM, Coleman MA, Richards MA and Rubens RD (1991) Weekly epirubicin for breast cancer with liver metastases and abnormal liver biochemistry. *Br J Cancer* **60**: 938–941

Twelves CJ, Dobbs NA, Michael Y, Summers LA, Gregory W, Harper PG, Rubens RD and Richards MA (1992) Clinical pharmacokinetics of epirubicin: the importance of liver biochemistry tests. *Br J Cancer* **66**: 765–769

© Cancer Research Campaign 1998